# Neural Oscillations in the Aging Brain Associated With Interference Control in Word Production

**DOI:** 10.1162/nol.a.15

**Published:** 2025-09-15

**Authors:** Xiaochen Y. Zheng, Vitória Piai

**Affiliations:** Donders Centre for Cognitive Neuroimaging, Radboud University, Nijmegen, The Netherlands; Cognitive Psychology Unit, Institute of Psychology, Leiden University, Leiden, The Netherlands; Donders Centre for Cognition, Radboud University, Nijmegen, The Netherlands

**Keywords:** aging, cognitive control, competition, mid-frontal theta, semantic interference, Stroop interference

## Abstract

Speaking is not only about retrieving words and structuring them into sentences; it also requires top-down control to plan and execute speech. In previous electrophysiological research with young adult speakers, mid-frontal theta oscillations have been observed using a picture–word interference paradigm. With this paradigm, participants name pictures while ignoring superimposed distractor words. In particular, mid-frontal theta power increases for categorically related distractors relative to other types of distractors, reflecting top-down interference control in resolving the competition between processing streams during word production. In the present study, we conceptually replicated the magnetoencephalography study by Piai, Roelofs, Jensen, et al. (2014) with an older group of healthy adults (mean age: 60 yr). Behaviorally, we replicated distractor semantic interference and Stroop-like interference effects typically observed in young adults. However, we did not find the corresponding theta modulation associated with these interference effects at the neural level. Instead, we found beta power decreases associated with interference control, mostly pronounced in the left posterior temporal and inferior parietal cortex. We further confirmed that these beta modulations were not present in the young adults’ data. The distinct spectro-spatial-temporal profile of the oscillatory effects in the older population may reflect different underlying dynamics relative to the midline frontal effect previously found in young adult speakers. Our results indicate that the neural underpinnings of top-down interference control may be modified by aging and that the mid-frontal theta cannot be the exclusive oscillatory pattern enabling interference control during spoken word production.

## INTRODUCTION

Speaking involves a set of processes that translate thoughts into words. It not only is about collecting the words and combining them into sentences or dialogues (Levelt, 1993; Levelt et al., 1999), but also requires sophisticated top-down control to plan and execute speech (Roelofs, 2021; Roelofs & Piai, 2011). In order to speak properly, one needs to plan and maintain the conversation goals and update the contents of working memory (Levelt et al., 1999; Martin & Slevc, 2014; Piai & Roelofs, 2013), to monitor what has been said and what is about to be said (Hartsuiker, 2014), to choose between different words, and to prevent interference from alternative words that get co-activated in the lexicon (Piai et al., 2013; Shao et al., 2015). Top-down control abilities, or in more general terms, cognitive control, change with advancing age—which impacts a wide range of cognitive domains, including working memory, attention, and inhibition (Braver & Barch, 2002; Paxton et al., 2008). Declines in the language domain, such as increased word-finding difficulties when speaking, are also commonly observed in older adults (Burke & Shafto, 2007; Mohan & Weber, 2019; Schmitter-Edgecombe et al., 2000; Valente & Laganaro, 2015). However, it is still unclear how aging affects the cognitive control process required for speaking.

Cognitive control in language production is commonly investigated using the picture–word interference task (Hermans et al., 1998; Lupker, 1979; Piai, Roelofs, Jensen, et al., 2014; Shitova et al., 2017). In such a task, participants name pictures while ignoring simultaneously presented written or spoken distractor words. The distractor words may be, for example, semantically related (e.g., a picture of an apple combined with the distractor word “banana”) or unrelated (e.g., picture apple, distractor word “bike”) to the target picture name or congruent with the target picture name (e.g., picture apple, distractor word “apple”). Naming performance, measured via response time (RT) or accuracy, changes as a function of the degree of relationship between the two competing streams (i.e., that of the picture and of the distractor). When the picture name and the distractor word are the same (i.e., congruent), their activations converge on a single word and further reduce the processing effort. By contrast, when the distractor word is incongruent with the target picture name, speakers need to inhibit the alternative word or enhance the target word (e.g., Piai, Roelofs, Jensen, et al., 2014; Roelofs, 2021; Shao et al., 2015) and prevent its interference, resulting in slower responses or more speech errors. Such a phenomenon is called Stroop-like interference, due to its shared nature with the classical Stroop effect (e.g., naming the red ink color of the printed word “blue”; Stroop, 1935). Conversely, when the distractor word is semantically related to the target picture name, it receives further activation from the picture and thus becomes a stronger competitor of the picture’s processing stream compared to a semantically unrelated distractor. Consequentially, the semantically related distractors cause larger delay and/or more errors than the unrelated ones, here referred to as semantic interference. This effect is well established in the language production literature (Bürki et al., 2020; Glaser & Düngelhoff, 1984; Lupker, 1979). Both the semantic interference and the Stroop-like interference effects can be considered to reflect the top-down cognitive control employed to deal with distracting stimuli, sometimes also termed *interference control* (e.g., Friedman & Miyake, 2004; Friehs et al., 2020; Piai et al., 2016).

Neuronal oscillations have been suggested to reflect the top-down interference control recruited for resolving the competition between processing streams during word production. In a magnetoencephalography (MEG) study, Piai, Roelofs, Jensen, et al. (2014) examined the neural mechanism underlying the competition between processing streams of the picture and the distractor word. A theta-power (4–8 Hz) increase was observed for the semantically related condition compared with the congruent condition (i.e., Stroop-like interference) roughly around 350–650 ms post stimulus onset. The order of power increase was analogous to the behavioral effects (i.e., related > congruent). This theta effect was localized to the superior frontal gyrus and postcentral gyrus, possibly also including the supplementary motor area and the anterior cingulate cortex. Similarly, theta power increases in the same time window were also found for the semantic interference effect (i.e., related vs. unrelated), source localized again to the superior frontal gyrus. Both effects were interpreted as reflective of different degrees of effort, or top-down interference control, in resolving the competition among the competing stimuli. Similar mid-frontal theta power increases have also been observed in other language production studies, particularly under conditions requiring more control due to stimuli interfering with production processes (Cui et al., 2024; Krott et al., 2019; Shitova et al., 2017; see Piai & Zheng, 2019, for a review on theta oscillation and cognitive control in language production). In the nonlinguistic literature, midline frontal theta oscillations, generated by the anterior cingulate cortex and superior frontal gyrus, are associated with working-memory load (Itthipuripat et al., 2013; Jensen & Tesche, 2002), performance monitoring (Cavanagh et al., 2012; Cohen, 2011; Luu et al., 2004), and increased top-down control to prevent interference (Cohen & Donner, 2013; Cohen et al., 2008; Hanslmayr et al., 2008; Nigbur et al., 2011).

Piai, Roelofs, Jensen, et al. (2014) argued that mid-frontal theta oscillations serve as the neural underpinning of top-down control needed for language production. However, their sample consisted only of young university students, whereas this conclusion might not extend to other groups, such as aging adults (Rad et al., 2018). For instance, language is a left lateralized function in the majority of the population but changes in lateralization may take place as part of compensatory or dedifferentiation processes in aging. The HAROLD (hemispheric asymmetry reduction in older adults) model has proposed that the prefrontal activity during cognitive performances tends to be less lateralized in older adults compared with young adults (Cabeza, 2002; but see Berlingeri et al., 2013). Moreover, the right hemisphere involvement is strongly associated with cognitive reserve (Brosnan et al., 2018), which may also play a role in top-down control in healthy aging. The age-related changes can also occur in the form of frequency-band shift. Mid-frontal theta power has been found to be significantly lower in older adults than in young adults during working memory tasks (Cummins & Finnigan, 2007; Kardos et al., 2014). Besides the changes in the theta dynamics with aging, higher frequency bands (e.g., beta or gamma) become more apparent while low-frequency bands (e.g., delta or theta) diminish with age (Werkle-Bergner et al., 2006). We wonder whether interference control in speaking changes over aging, accompanied by frequency changes. How would neural oscillations, and in particular, the theta dynamics associated with the competition between processing streams in word production change for older adults?

In the present study, we perform a conceptual replication of Piai, Roelofs, Jensen, et al. (2014) with an older population, with the particular interest in how the mid-frontal theta is modulated by interference control, as measured through the semantic interference and the Stroop-like interference effects. We expect that the older population may differ from young adults in the neural signature of cognitive control due to aging. For example, differences might manifest as changes in amplitude, spatial distribution, or spectral characteristics (Brosnan et al., 2018; Cabeza, 2002; Werkle-Bergner et al., 2006).

## MATERIALS AND METHODS

### Participants

A recent study on word production has shown that age-related change in neurophysiological activity emerges from the age of 40 (Krethlow et al., 2024). In our study, we collected data from a group of participants around the age of 60 to gain a broader insight into how an older brain controls language production and prevents interference. Twenty-five participants from an older population (mean age = 61.5; range 46–75, 15 men, 10 women) than in Piai, Roelofs, Jensen, et al. (2014) took part in the study for monetary compensation. All of them were native speakers of Dutch, right-handed, with normal or correct-to-normal vision. The study was conducted in accordance with the Declaration of Helsinki, was approved by the local ethics committee (Arnhem-Nijmegen CMO, NL58437.091.17), and all participants provided written informed consent. Nine participants’ data were excluded from the analyses due to (1) excessive metal-related artifacts in the MEG (*N* = 5), (2) technical problems in the MEG (*N* = 2), and (3) too few correct responses (*N* = 2). This left a final sample of 16 participants (Mean age = 59.2, range = 46–72, 9 men, 7 women).

In addition, we retrieved the preprocessed behavioral and MEG data of the young adult group from the original study (Piai, Roelofs, Jensen, et al., 2014). This concerns 17 healthy right-handed adults (mean age = 21.8, *SD* = 3.56, 6 men). We included these data for better comparison of the age-related effects.

### Materials, Design, and Behavioral Procedure

**Figure 1.**
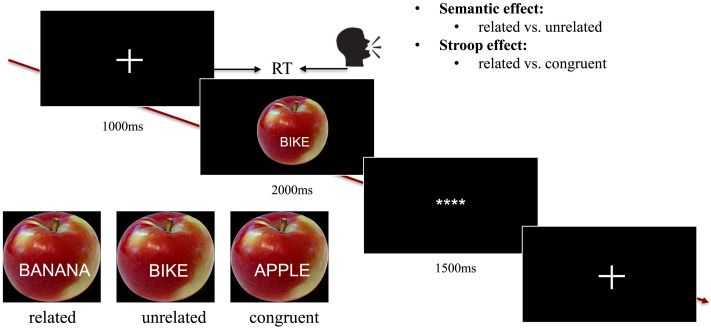
Experimental paradigm and conditions. The actual experimental stimuli are in the native language of the participants.

For testing the older adults, 88 color pictures were taken from the BOSS database (Brodeur et al., 2010), forming 16 different semantic categories with five to six objects pertaining to each category. For each picture, distractor words were the picture name (i.e., congruent), from the same semantic category as the picture (i.e., related), or from a different semantic category (i.e., unrelated; Figure 1). Thus, all distractor words belonged to the response set. We made sure that the related and unrelated distractors were phonologically unrelated to the target picture. All participants saw each picture once in each condition. The picture–word trials were randomized using Mix (van Casteren & Davis, 2006), with one unique list per participant. In this way, the order of appearance of a given item in a given condition was counterbalanced across participants. Participants saw no more than five consecutive trials from the same condition, and there was no consecutive repetition of the distractor words or target pictures from trial to trial. Participants were instructed to name the picture and to ignore the distractor word. Both speed and accuracy were emphasized.

The experiment was run using the software Presentation (Version 18.0; Neurobehavioural Systems, 2025). The background color of the computer screen was set to black, with a resolution of 1920 × 1080 pixels, at a refresh rate of 60 Hz. Distractor words were presented in white, centered on the picture. Trials began with a fixation cross presented for 1 s, followed by the presentation of the picture–word stimulus for 2 s. Then an intertrial interval of 1.5 s was presented with **** on the screen (Figure 1). Participants’ responses were coded online as correct (i.e., identical to the target picture name) or incorrect. The experiment started with six practice trials. Participants were familiarized with the pictures and their names during the MEG preparation.

For the young adult data received from the original study (Piai, Roelofs, Jensen, et al., 2014), 36 line drawings of common objects (belonging to nine different semantic categories) from the picture database of the Max Planck Institute for Psycholinguistics were used. Each trial began with a fixation cross for 1.75 s, followed by the stimulus for 1.5 s. Three asterisks followed, indicating a blinking moment for 1.5 s, followed by an empty screen for 0.5 s. The rest of the experimental setup was the same as the current study.

### MEG Acquisition

MEG data were acquired with a 271 axial gradiometer system (CTF Systems Inc., VSM MedTech Ltd.). The signals were analog low-pass filtered at 300 Hz and digitized at a sampling rate of 1200 Hz. Each participant was positioned in the MEG chair with pillows as they preferred. Three localization coils were attached to the participant’s head (the nasion, and the left and right ear canal). Throughout the measurement, head position was continuously monitored using custom software (Stolk et al., 2013). Participants’ head positions were readjusted during the breaks to maintain the original position (max. 5 mm away). Four bipolar Ag/AgCl electrode pairs were used to measure the horizontal and vertical electrooculograms (EOG), the mouth electromyogram (EMG), and the electrocardiogram (ECG). Electrode impedance was kept below 20 kΩ. The MEG session lasted approximately 1 hr, including preparation time.

Structural T1-weighted magnetic resonance imaging (MRI) scans of participants’ heads were acquired from a 3T Siemens scanner, either immediately after the MEG session or on a different day, no more than 4 weeks apart.

MEG data from the original study (Piai, Roelofs, Jensen, et al., 2014) were recorded using the same MEG system, with the same low-pass filter and sampling rate. Structural MRIs were acquired with a 1.5 T Siemens system.

### Behavioral Data Analysis

Picture naming responses were recorded time locked to the picture onset, and RTs were manually calculated offline using the speech analysis program Praat (Boersma & Weenink, 2016), blinded to conditions. Note that for the original study by Piai, Roelofs, Jensen, et al. (2014), naming responses were evaluated and RTs were calculated in real time but not corrected offline. Responses containing disfluencies or errors were coded as invalid. For both datasets, responses containing incorrect picture names or disfluencies were considered as errors and excluded together with technical errors from the subsequent RT and MEG analyses.

The statistical analyses of the behavioral data were performed with linear mixed-effects models using the lme4 package (Version 1.1.25; Bates et al., 2015) and lmerTest package (Kuznetsova et al., 2017) in R (Version 4.0.2; R Core Team, 2020). RTs were log-transformed to reduce skewness and approach a normal distribution. We used a model of RTs as a function of condition (related vs. unrelated vs. congruent, with the related condition as the reference), group (older adults vs. young adults), and their interaction (Condition × Group), with a by-participant random slope for condition. We then explored the difference between conditions using the same model for each age group. Analysis of speech errors was done using a generalized mixed-effects model (binomial family). Due to singular fitting of the full model, we only included random intercept for participants.

### MEG Data Analysis

#### Preprocessing

We performed all MEG analyses using the Fieldtrip open source MATLAB toolbox (Oostenveld et al., 2011) and custom analysis scripts in MATLAB R2018b (Version 9.5; MathWorks, 2018). We first segmented the continuous MEG and EEG (i.e., EOG, EMG, and ECG) data into epochs from 500 ms before to 1,000 ms after the target picture onset. The data were demeaned using the 500 ms interval before picture onset, down-sampled to 600 Hz, and then low-pass filtered with a cutoff of 55 Hz. EOG artifacts (i.e., eye blinks and saccades) as well as unambiguous speech artifacts were removed using independent component analysis, facilitated by visual inspection of the EEG data. Trials with remaining artifacts and/or bad sensors were rejected through visual inspection of the MEG data. This artifact rejection procedure was done before trials were separated by condition. To prevent contamination of the signal with speech-related artifacts, only trials with RTs longer than 700 ms were included.

There were a few differences in the preprocessing pipelines between the present study (older adults) and the young adult group from the original study (Piai, Roelofs, Jensen, et al., 2014). Specifically, no low-pass filter was applied and no independent component analysis was used for artifact rejection in the young adult data. These differences reflect the preprocessing standards at the time but are unlikely to introduce confounds in the effects of interest. Importantly, these preprocessing differences do not impact the time-resolved spectral decomposition (which was the same for both datasets) in any particular way that would systematically affect the differences across conditions between the two groups.

#### Sensor-level analysis

Synthetic planar gradients were computed for subsequent time-frequency representation (TFR) analysis (Bastiaansen & Knösche, 2000). The TFRs were computed using the same protocol for both age groups: between 500 ms pre to 1 s post stimulus onset, at frequencies from 2 to 30 Hz, with a sliding time window of three cycle’s length, advancing in steps of 10 ms and 1 Hz. Each time window was multiplied with a Hanning taper. TFRs were baseline corrected using the 500 ms interval before stimulus onset, based on the normalized difference between the signal of interest and its baseline (i.e., S − B/(S + B)).

#### Source-level analysis

To examine the source of effects found in the sensor-level analysis, we applied a frequency-domain beamforming technique (dynamic imaging of coherent sources, or DICS; Gross et al., 2001) to all the sensor data. Individual volume conduction models were computed using a realistic single-shell model (Nolte, 2003). The required brain–skull boundary was obtained from the participant-specific T1-weighted anatomical images, aligned to the CTF MEG coordinate system. To normalize across participants, we warped the CTF-aligned MRI scans to the Montreal Neurological Institute (MNI) space template to obtain participant-specific source model grids, with 10 mm resolution. Using the volume conduction models, lead field matrices were computed for each grid point for individual participants. Foreshadowing the results, sensor-level effects were not found in the 4–8 Hz frequency range, but rather in the 16–30 Hz range. This range formed the basis for the source-level analysis. The cross-spectral density matrix was computed at a central frequency of 23 Hz with 7 Hz smoothing, resulting in a frequency range of 16–30 Hz. We computed a common spatial filter for both conditions combined together with their corresponding pre-stimulus baseline windows, and then applied the spatial filter to the Fourier transformed sensor-level data per condition to estimate source-level power for each grid point. The acquired sources were baseline corrected using corresponding sources for the baseline windows (i.e., a time window with the same length before the target picture onset), in the same way as the sensor-level baseline correction (i.e., S − B/(S + B)). For visualization, the source-level results were interpolated to a template anatomical MRI.

#### Statistical testing

The statistical analysis of the MEG data was run using a nonparametric cluster-based permutation test (Maris & Oostenveld, 2007). We compared two contrasts of interest, that is, semantic interference effect (related vs. unrelated) and Stroop-like effect (related vs. congruent). This test provides a cluster-based *p* value (family-wise error corrected) of adjacent time points, sensors, and frequencies that exhibit similar differences across conditions. The permutation distribution was constructed by randomly partitioning the original data 1,000 times. We considered spectro-spatial-temporal clusters with their cluster-level statistic corresponding to a *p* value smaller than 0.05 to be significant.

Based on previous findings (Piai, Roelofs, Jensen, et al., 2014), we constrained the analyses of the theta band effect to the time window of 350 to 650 ms post stimulus onset and to all frontal and central MEG sensors (i.e., sensors labeled MLF*, MRF*, MLC*, MRC*). Between-group comparison was performed using the same, hypothesis-driven selection of sensors, frequency range, and time window. Specifically, the contrasts of interest (e.g., related vs. unrelated) were extracted first for each age group, then compared between groups using the permutation test. For the broader band exploratory analysis in the older adult group, we used the entire time window (i.e., 0–700 ms post stimulus onset) and all available MEG sensors. We further explored the beta band effect in the young adult group in a post hoc analysis, based on the effect observed in the older adult group (200–600 ms, all left central and parietal MEG sensors).

## RESULTS

### Picture Naming Performance

**Figure 2.**
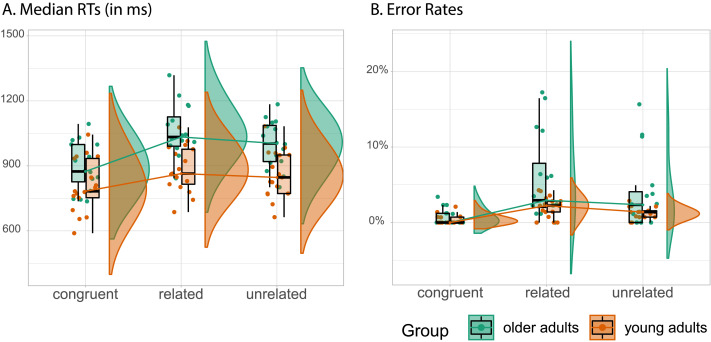
Raincloud plots of participants’ median response times (RTs) and error rates for the three experimental conditions (related vs. unrelated vs. congruent). (A) RTs. (B) Error rates. The outer shapes represent the distribution of the data over participants, the thick horizontal line inside the box indicates the group median, and the bottom and top of the box indicate the group-level first and third quartiles of each condition. Each dot represents one participant. The young adult data were obtained as preprocessed data from Piai, Roelofs, Jensen, et al. (2014).

Figure 2 shows the picture naming performance for both the older and the young adult groups, including their median naming RTs (Figure 2A) and their error rates (Figure 2B) for each experimental condition.

While the young adults were consistently faster than the older group in all conditions (*β* = −0.18, *SE* = 0.04, *t* = −4.59, *p* < .001), both groups showed the expected semantic interference effect and Stroop-like effect in RTs. For the older adult group, participants were slower in the related condition (the mean of individual participants’ median RTs: Mean = 1,054, *SD* = 127) than in the unrelated condition (Mean = 1,000, *SD* = 109), showing a significant semantic interference effect (*β* = 0.04, *SE* = 0.01, *t* = 4.56, *p* < .001). They were also slower in the related compared with the congruent condition (Mean = 894, *SD* = 112), showing a significant Stroop-like effect (*β* = 0.16, *SE* = 0.02, *t* = 8.67, *p* < .001). The same pattern was observed for the young group (Mean_related_ = 885, *SD*_related_ = 106; Mean_unrelated_ = 861, *SD*_unrelated_ = 109; Mean_congruent_ = 808, *SD*_congruent_ = 124): their RTs showed both the semantic interference effect (*β* = 0.02, *SE* = 0.006, *t* = 3.95, *p* = .001) and the Stroop-like effect (*β* = 0.10, *SE* = 0.01, *t* = 8.88, *p* < .001).

Due to differences in experimental setup between the two datasets, directly comparing the raw magnitudes of interference effects is not straightforward. To enable meaningful comparisons, we normalized both the semantic interference effect (i.e., (related − unrelated)/unrelated) and Stroop-like effect (i.e., (related − congruent)/congruent) for each participant and then compared the effects between the two age groups. Results showed that the semantic effects did not differ between groups (Mean_old_ = 0.054, *SD*_old_ = 0.047, Mean_young_ = 0.028, *SD*_young_ = 0.025, *t*(22.4) = 1.95, *p* = .06). In contrast, the Stroop-like effect was significantly larger in the older adult group (Mean_old_ = 0.184, *SD*_old_ = 0.098) than the young adult group (Mean_young_ = 0.102, *SD*_young_ = 0.057, *t*(23.8) = 2.93, *p* = .007).

Similar results were found for error rates: While the young adults made fewer errors than the older adults (*β* = −0.89, *SE* = 0.35, *z* = −2.56, *p* = .011), both groups showed the expected semantic and Stroop-like effects. For the older adult group, participants made more naming errors for related (Mean = 5.8%, *SD* = 5.6%) compared with the unrelated condition (Mean = 3.7%, *SD* = 4.9%), showing a significant semantic interference effect (*β* = 0.43, *SE* = 0.18, *z* = 2.44, *p* = .015). They also made more errors for related compared with the congruent condition (Mean = 0.7%, *SD* = 1.1%), showing a significant Stroop-like effect (*β* = 2.05, *SE* = 0.30, *z* = 6.86, *p* < .001). The same held for the young group (Mean_related_ = 2.1%, *SD*_related_ = 1.4%; Mean_unrelated_ = 1.3%, *SD*_unrelated_ = 0.8%; Mean_congruent_ = 0.4%, *SD*_congruent_ = 0.6%; semantic interference effect: *β* = 0.50, *SE* = 0.24, *z* = 2.08, *p* = .037; Stroop-like effect: *β* = 1.58, *SE* = 0.35, *z* = 4.53, *p* < .001). We did not compare the normalized interference effects in error rate between groups: Since many participants made zero errors in the congruent condition, it was difficult to assess the relative scores.

### Planned TFR Analysis

We first investigated whether the mid-frontal theta effects observed in the young group can be replicated in the older group for both the semantic contrast and the Stroop-like contrast.

#### Semantic interference effect (related vs. unrelated): No mid-frontal theta in the older group

**Figure 3.**
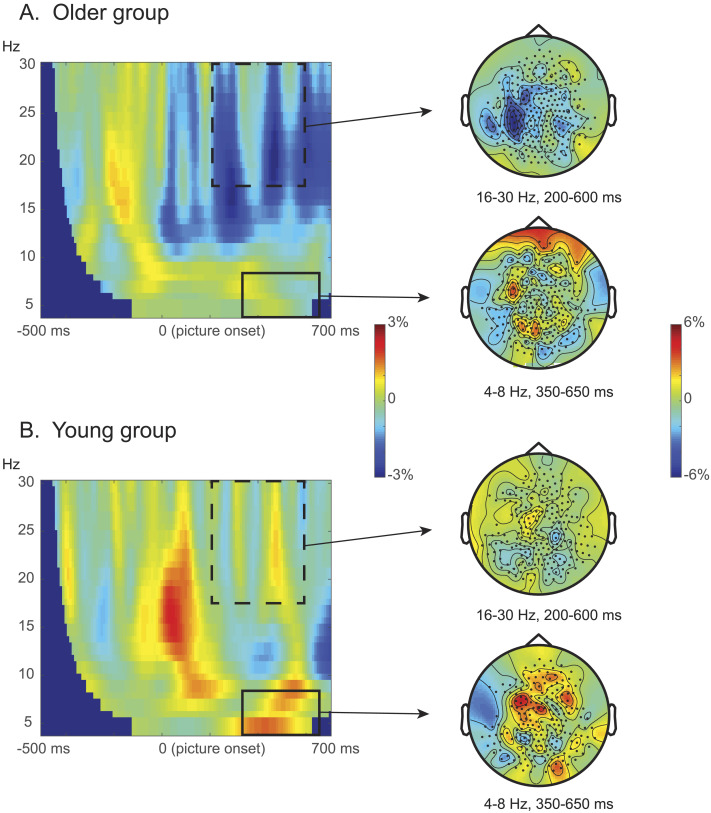
Semantic interference effects. (A) Older adults. (B) Young adults. Left panels: Stimulus-locked time-resolved spectrum of the contrast between related versus unrelated conditions, averaged over all sensors. Right panels: Topography of the semantic contrast (i.e., related vs. unrelated) in the beta band (16–30 Hz) between 200 to 600 ms post picture onset, and in the theta band (4–8 Hz) between 350 and 650 ms post picture onset. The solid rectangles highlight the clusters used for the planned time-frequency representation (TFR) analysis, and the dotted rectangles highlight the clusters that were most pronounced in the exploratory TFR analysis. Color bars indicate the relative power change between conditions. The young adult data were obtained as preprocessed data from Piai, Roelofs, Jensen, et al. (2014).

Figure 3 shows the contrasts between related and unrelated distractors for both the older adults (Figure 3A) and the young adults (Figure 3B), presented as the power differences between related and unrelated conditions in the 4–30 Hz range between −500 and 700 ms time locked to the picture onset. As reported in the original study by Piai, Roelofs, Jensen, et al. (2014), there was a significant difference in the young group between the related and unrelated conditions in the theta band (4–8 Hz), between 350 and 650 ms post picture onset for the fronto-central sensors (*p* = .026). However, this semantic-related theta effect was not significant in the older adult group (*p* = .490). The semantic-related theta effects did not significantly differ between the age groups (*p* = .102).

#### Stroop-like effect (related vs. congruent): No mid-frontal theta in the older group

**Figure 4.**
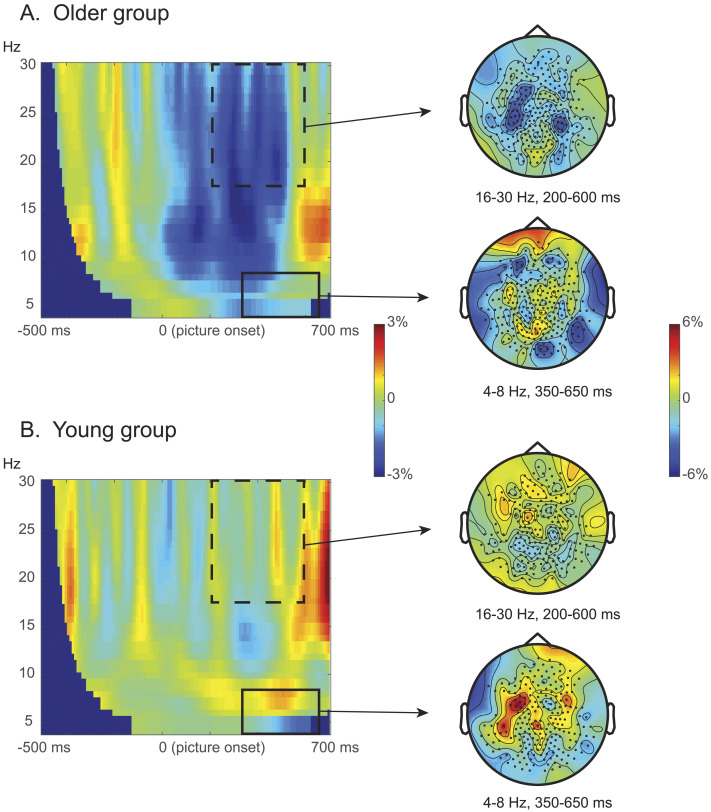
Stroop-like effects. (A) Older adults. (B) Young adults. Left panels: Stimulus-locked time-resolved spectrum of the contrast between related versus congruent conditions, averaged over all sensors. Right panels: Topography of the stroop-like contrast (i.e., related vs. congruent) in the beta band (16–30 Hz) between 200 and 600 ms post picture onset, and in the theta band (4–8 Hz) between 350 and 650 ms post picture onset. The solid rectangles highlight the cluster used for the planned TFR analysis, and the dotted rectangles highlight the clusters that were most pronounced in the exploratory TFR analysis. Color bars indicate the relative power change between conditions. The young adult data were obtained as preprocessed data from Piai, Roelofs, Jensen, et al. (2014).

Figure 4 shows the Stroop-like contrasts for both the older adult (Figure 4A) and the young adult (Figure 4B), presented as the power differences between related and congruent conditions in the 4–30 Hz range between −500 and 700 ms time locked to the picture onset. Again, as reported in the original study (Piai, Roelofs, Jensen, et al., 2014), there was a significant difference in the young adult group between the related and congruent conditions in the theta band (4–8 Hz), with the effect being between 350 and 650 ms post picture onset in the fronto-central sensors (*p* = .044). In contrast, this Stroop-like theta effect was not presented in the older adult group (*p* = 1). The Stroop-like effects significantly differed between the age groups (*p* = .046).

### Exploratory TFR Analysis

Interestingly, while both predicted theta effects were absent in the older adult group, visual inspection suggested a difference in the beta band (Figures 3 and 4). These beta-related effects seemed very similar in terms of time window and frequency spectrum, for both the semantic and the Stroop-like contrasts. To explore these potential effects, we applied a cluster-based permutation test to all the sensors available in the full bandwidth (4–30 Hz) and the entire time window (0–700 ms).

The comparison between the related and unrelated conditions in the older adult group showed that there was indeed a significant difference in the beta band (*p* = .026). The effect was mostly pronounced around 200–600 ms post target picture onset, clustered around 16–30 Hz. The topographical map shows that the effect was mostly salient at the left central and parietal sensors. In contrast, we did not find this beta effect in the young adult group (*p* = 1).

Similar to the semantic-related effect, a visible difference in the TFR between the related and congruent conditions in the beta band was observed in the older adults. However, this Stroop-like interference effect did not reach statistical significance (*p* = .056) in the cluster-based permutation test. Notably, this beta effect is entirely absent in the young adult group (*p* = 1).

#### Source localization of the semantic-related beta effect

**Figure 5.**
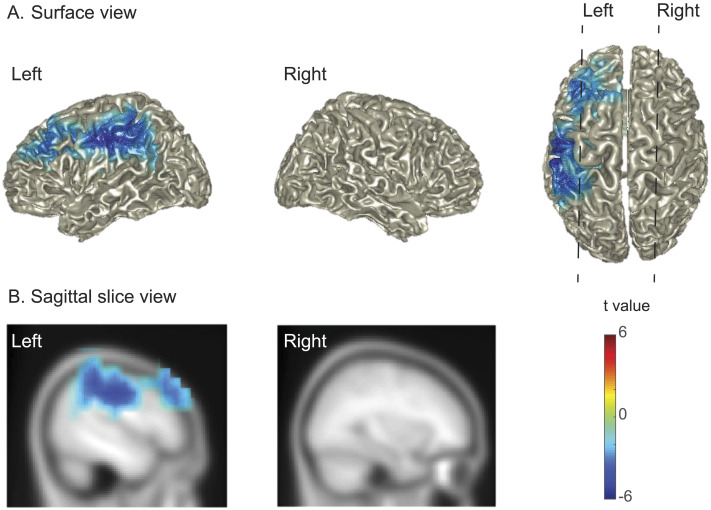
Source localization of the semantic-related beta effect in the older adult group. (A) Power difference between 16 and 30 Hz for the semantic interference effect (i.e., related vs. unrelated) in the time window of 200 to 600 ms post picture onset. (B) Two representative sagittal slices from the left and right hemisphere, corresponding to the dashed lines in A. The color bar indicates the *t* values for individual grid points. Only grid points associated with the significant cluster are colored.

To further understand the semantic-related beta effect observed in the older adult group, we performed source analyses in the beta band (16–30 Hz) for the semantic interference contrast (time window of 200–600 ms). The results are shown in Figure 5. The beta power decreases associated with the semantic interference effect were found exclusively in the left hemisphere. These were distributed around the pre- and post-central gyrus, supramarginal gyrus, and inferior parietal cortex, according to the automated anatomical labeling (AAL) template (Tzourio-Mazoyer et al., 2002).

## DISCUSSION

To investigate age-related differences in interference control during spoken word production and the associated changes in neuronal oscillations, we conceptually replicated the MEG study by Piai, Roelofs, Jensen, et al. (2014) in a group of aging adults with an average age of 60. Using a picture–word interference task, we examined how mid-frontal theta—widely considered a marker of top-down cognitive control—is modulated by different types of interference. Behaviorally, the older adults displayed a pattern consistent with that commonly observed in young adults: Speakers were better at naming pictures with congruent than with semantically related distractors, replicating the Stroop-like interference effect (Piai et al., 2020; Piai, Roelofs, Jensen, et al., 2014; Shitova et al., 2017); they were also better at naming pictures with semantically unrelated compared to related distractors, replicating the semantic interference effect (Krott et al., 2019; Lupker, 1979; Piai et al., 2020; Piai, Roelofs, Jensen, et al., 2014; Schriefers et al., 1990). These findings align with previous studies investigating picture–word interference in older populations (Graf et al., 1995; Rizio et al., 2017; Taylor & Burke, 2002).

Neurally, however, the older adults exhibited a very different profile compared with young participants in the original study by Piai, Roelofs, Jensen, et al. (2014) and other picture–word interference studies (Krott et al., 2019; Shitova et al., 2017). Unlike studies involving young adults (mean age 22–23 yr), we did not observe a mid-frontal theta effect for either the Stroop-like or the semantic interference conditions. Instead, we found a decrease in beta power associated with both interference effects, which was absent in the young adult data from the original MEG study by Piai et al. Importantly, while the beta power decrease was visible for both interference effects in older adults, the decrease associated with the Stroop-like effect was not statistically significant. Crucially, neither the semantic-related nor the Stroop-like beta effects were present in the young adult group.

The theta power increases observed in picture–word interference tasks have been associated with top-down control in preventing the interference from the distractor words (Krott et al., 2019; Piai, Roelofs, Jensen, et al., 2014; Piai & Zheng, 2019; Shitova et al., 2017). The lack of a theta effect in this group of aging adults lends multiple interpretations. First—using a reverse-inference logic—it could suggest decreased abilities of detecting or suppressing interference in the older adults. It has been proposed that the attention-related prefrontal cortex function is altered with aging, leading to a decline in inhibitory control (Chao & Knight, 1997; West, 1996; but see, e.g., Kramer et al., 1994). For example, research has shown less theta power increase in older adults compared with younger adults during selective memory retrieval, suggesting the older adults are less capable of detecting memory interference (Ferreira et al., 2019). Nevertheless, the prolonged naming times instead of incorrect naming responses in most of the trials suggest that our older participants were still able to inhibit the irrelevant information and resolve the competition between the streams of processing the picture and the distractor. Therefore, the hypothesized deficit in inhibition, if true, is limited to the neural level and not yet hampering performance on the behavioral level. Second, more recent theories of cognitive aging emphasize that the prefrontal cortex may be not only a major source of dysfunction but also a source of compensation (McDonough et al., 2013). A functional MRI study on picture–word interference has reported that semantically related distractors led to increased recruitment of regions including the left middle frontal gyrus in older adults compared with the young adults (Rizio et al., 2017). It could be that our older adults instantly exhibit high-level (mid-frontal) theta activities for all the conditions due to (over-)compensation, resulting in a missing difference in the theta band between conditions. However, this assumption is unlikely to hold in our data, as the induced mid-frontal theta observed in the previous study of young adults is absent across all three experimental conditions in the older adult group (Figure S1 in the Supporting Information, available at https://doi.org/10/1162/nol.a.15). Third, it could be that the mid-frontal theta only reflects the top-down cognitive control of the young adult brains but not of the older adult brains, given that our older participants have clearly shown behavioral effects of interference control. Instead, interference control, at least in the older adult brains, can come about via other neural signatures than frontal theta oscillations (e.g., degeneracy).

Building on the previous explanation, could the beta effect we observed in older adults be the result of a frequency band shift from the theta effect observed in young adults? This seems highly unlikely. Beyond the opposite polarity (i.e., power increase vs. decrease for the same effect), the neural signatures in the current study and those in Piai, Roelofs, Jensen, et al. (2014) are spatio-temporally distinct. The theta increase is relatively short-lived, clustered around the 350–650 ms time window, while the beta decrease is more widespread and starts earlier in the processing. Moreover, the interference-related theta power modulations exhibit a fronto-central topography, commonly identified as mid-frontal theta—a well-established marker of cognitive control (Cavanagh et al., 2012; Cohen, 2011; Hanslmayr et al., 2008; Nigbur et al., 2011). In contrast, the beta power modulations associated with the Stroop-like and the semantic interference effects in the present study display a more left-lateralized, centro-parietal profile, likely reflecting a language-related mechanism. Notably, beta-band effects have been previously reported in picture–word interference studies (e.g., Krott et al., 2019). However, the beta modulation in those studies exhibit a different spatio-temporal profile compared to our findings in older adults. Specifically, the beta effect reported by Krott et al. was observed in fronto-central electrodes and within an earlier time window (approximately 50–100 ms post stimulus onset). This divergence suggests that the two beta-band signatures reflect distinct neural mechanisms.

A more intriguing question, then, is what this new beta can tell us about interference control during spoken word production. Before discussing its possible functionality, a first suspect we need to exclude is motor preparation. Motor-related activity is well characterized by power decreases in the beta band in sensorimotor areas, typically in the range of 15–30 Hz (Cheyne, 2013; Pfurtscheller & Lopes da Silva, 1999). In language production, beta-band power decreases are observed prior to and during speech in motor regions associated with speaking (Salmelin et al., 1995, 2000; Salmelin & Sams, 2002). Nevertheless, the beta power decreases we have observed are unlikely to be motor-related, or RT-related. In that case, one would expect an earlier onset of beta power decreases for the congruent or unrelated condition (i.e., shortest RTs) than the semantically related condition (i.e., longest RTs). This would result in a neural effect in the opposite direction of what we observed (i.e., beta power increase instead of decrease). Alternatively, the beta effect could be related to working memory gating for task-relevant versus irrelevant information (Limanowski et al., 2020; Spitzer & Haegens, 2017) or semantic memory retrieval (Hanslmayr et al., 2009; Piai & Zheng, 2019). Alpha-beta power decreases in posterior temporal and inferior parietal brain areas have been reliably found in tasks that require conceptually driven word production, such as context-driven word production (Piai et al., 2015, 2018; Piai, Roelofs, & Maris, 2014), picture naming (Grappe et al., 2019), and verb generation (Pang et al., 2011). It is considered to reflect different degrees of retrieval of conceptual and lexical information from memory. The present results of beta power decreases resemble the spatial pattern associated with context-driven word production (see, e.g., Figure 5 in Roos & Piai, 2020). The similar recruitment of left temporal-parietal brain regions suggests a potential shared mechanism related to semantic memory retrieval. However, this claim begs the question of why the beta effect is absent in the young adult population. At present, we do not have a definitive explanation. One possibility is that the beta power decreases are related to task demand. For young adults, word retrieval across all three conditions may be relatively effortless, resulting in no observable difference in beta oscillations between conditions. Older adults, by contrast, might resolve the interference at the semantic and/or lexical levels, without relying heavily on interference control. However, these explanations are highly speculative and warrant further investigation.

### Limitations

Besides the inconclusive functional explanation of the beta modulation observed in the older adult group, there are several other limitations of the study that we would like to address. First, beta-range modulations were observed in the older adults for both experimental contrasts associated with interference control; however, only the semantic interference effect reached significance. This indicates that the observed pattern was not stable enough to consistently replicate across both effects, which are thought to reflect the same interference control process. This inconsistency may result from the limited sample size, which reduces statistical power, or it might point to fundamental differences between these two control processes, as operationalized by these different contrasts. Further research is required to clarify this issue. Second, our findings indicate that the “no longer young” brain controls language production differently than in young adults. However, given the wide age range of participants in the older adult group (Figure S2), individual differences may exist within this group (see Figure S3 and Figure S4 for visualization of key effects following a median split of the older adult group). This variability in age, which likely entails age-related changes in cognitive control, may partially explain why the Stroop-like beta effect was not significant in the older adult group. We hope that future large-scale, well-powered studies will explore these individual differences. Third, while both interference effects typically observed in young adults were replicated behaviorally in the older adults, the Stroop-like RT effect was larger in the older adult group, potentially reflecting some age-related difference in terms of efficiency of control. However, this between-group difference was not observed for the semantic interference effect. Finally, although none of the young or older adults we tested were simultaneous bilinguals, many spoke a second or a third language (e.g., English or German). Given that bilingualism can influence the neural functioning of cognitive control (Carter et al., 2023; DeLuca et al., 2019; Gold et al., 2013), it is possible that this factor contributes to the observed group difference, especially considering that second language proficiency may vary between the two groups due to age differences. These limitations highlight the need for future research to investigate interference control processes in speaking across age groups.

### Conclusion

In summary, with an older group of healthy adults, we replicated the behavioral Stroop-like interference and semantic interference effects commonly found in young adults, but not the corresponding theta modulation on the neural level (cf. Krott et al., 2019; Piai, Roelofs, Jensen, et al., 2014; Shitova et al., 2017). Instead, we found beta power decreases associated with these interference effects, with, specifically, the semantic interference effect localized to the left posterior temporal and inferior parietal cortex. The distinct spectro-spatial-temporal profile of the oscillatory effects in the older population may reflect different underlying dynamics other than the mid-frontal theta effect. Such dissociation between behavior and neural activity has also been shown in a recent study (Krethlow et al., 2024), where age-related changes at the neurophysiological level starts from the age of 40 and the behavioral difference only appears after 70. Our findings suggest that the mid-frontal theta cannot be the exclusive oscillatory pattern enabling interference control during spoken word production.

## ACKNOWLEDGMENTS

We thank Maaike ten Buuren and Rosemarije Weterings for help with data collection and behavioral data analysis.

A research consortium grant was awarded by the Netherlands Organization for Scientific Research to the Language in Interaction Consortium (024-001-006). The authors were members of the research consortium.

## FUNDING INFORMATION

Xiaochen Y. Zheng, Nederlandse Organisatie voor Wetenschappelijk Onderzoek (https://dx.doi.org/10.13039/501100003246), Award ID: VI.Veni.231C.010. Vitória Piai, Nederlandse Organisatie voor Wetenschappelijk Onderzoek (https://dx.doi.org/10.13039/501100003246), Award ID: 451-17-003.

## AUTHOR CONTRIBUTIONS

**Xiaochen Zheng**: Conceptualization: Supporting; Formal analysis: Lead; Investigation: Lead; Project administration: Lead; Visualization: Lead; Writing – original draft: Lead; Writing – review & editing: Lead. **Vitória Piai**: Conceptualization: Lead; Data curation: Lead; Funding acquisition: Lead; Supervision: Lead; Writing – original draft: Supporting; Writing – review & editing: Supporting.

## DATA AND CODE AVAILABILITY STATEMENTS

Data and codes are available at the Donders repository (https://doi.org/10.34973/6sa5-0t15).

## Supplementary Material



## TECHNICAL TERMS

Picture–word interference task: A task where people name pictures while ignoring distracting words; used to study language production.
Magnetoencephalography (MEG): A neuroimaging technique that measures brain activity by detecting the magnetic fields produced by the electrical currents in the brain.
Neural oscillations: Refers to rhythmic electrical activity in the brain that reflects communication between different brain areas.
Dynamic imaging of coherent sources (DICS): A method to reconstruct the sources in the brain that generated the activity measured over the scalp.
Cluster-based permutation test: A statistical method that finds significant effects in complex datasets by comparing observed data to random permutations.
