# Supplementary material for: Neural Oscillations in the Aging Brain Associated With Interference Control in Word Production
Source: Neurobiol Lang (Camb). 2025 Sep 15;6:nol.a.15. doi: 10.1162/nol.a.15 (PMC12459976; doi:10.1162/nol.a.15)
Supplement: Supplementary file 1 [file nol-6-1-15-s001.pdf]

Supplementary materials

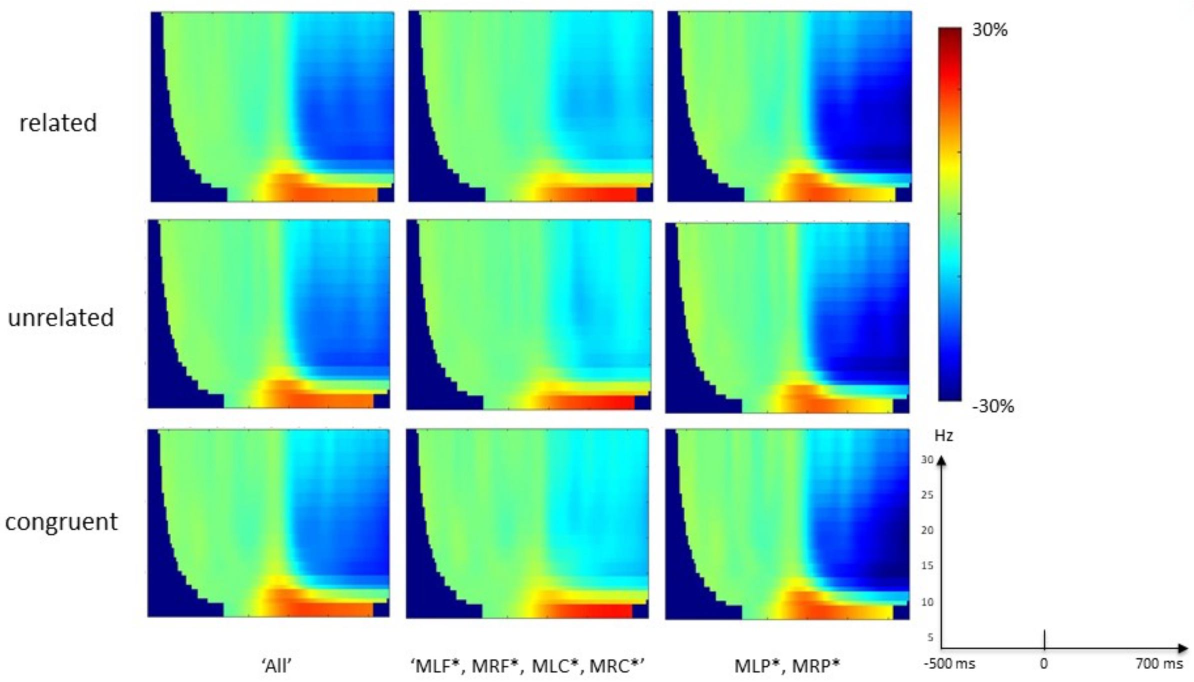

Figure S1. Time-frequency representation of the three experimental conditions for the older adults group, averaged across all sensors (left panel), fronto-central sensors (middle panel), or parietal sensors (right panel). All conditions are baseline corrected by the 500 ms time window before picture onset. Unlike Piai et al. (2014), data in the current study have only shown evoked theta power, regardless of experimental conditions and spatial profiles.

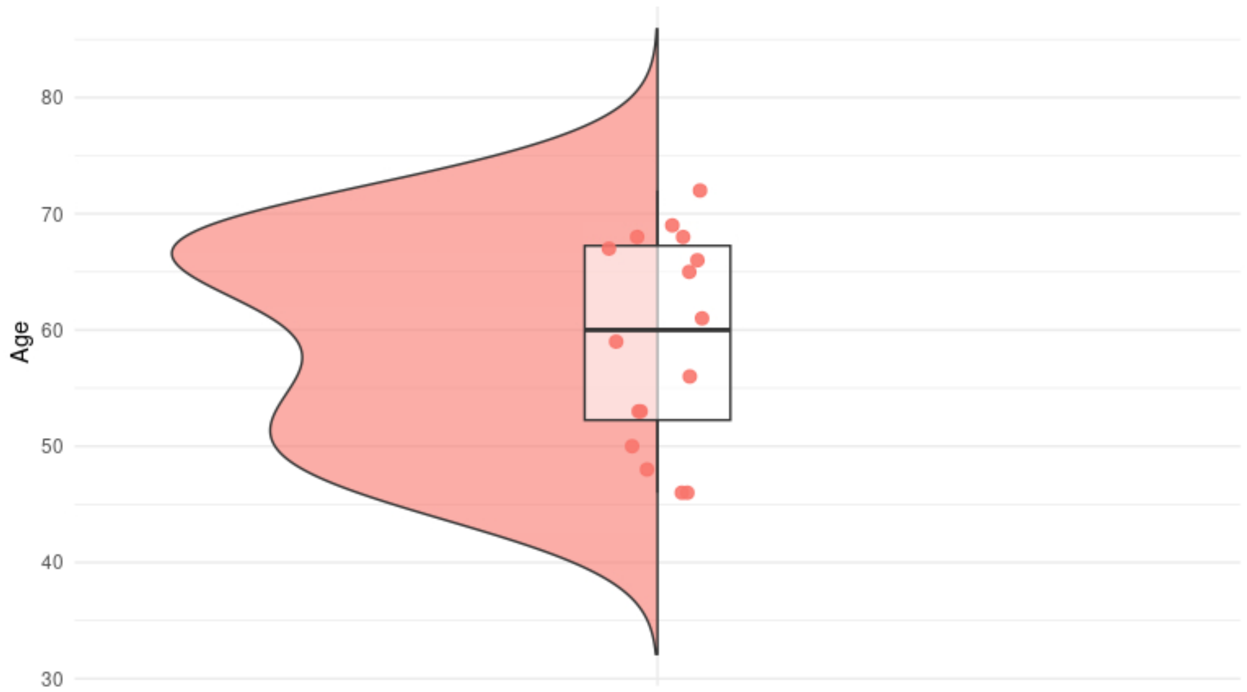

Figure S2. Age distribution of the older adult group (N = 16). The outer shapes represent the distribution of the age of all participants, the thick horizontal line inside the box indicates the group median, and the bottom and top of the box indicate the group-level first and third quartiles. Each dot represents one participant.

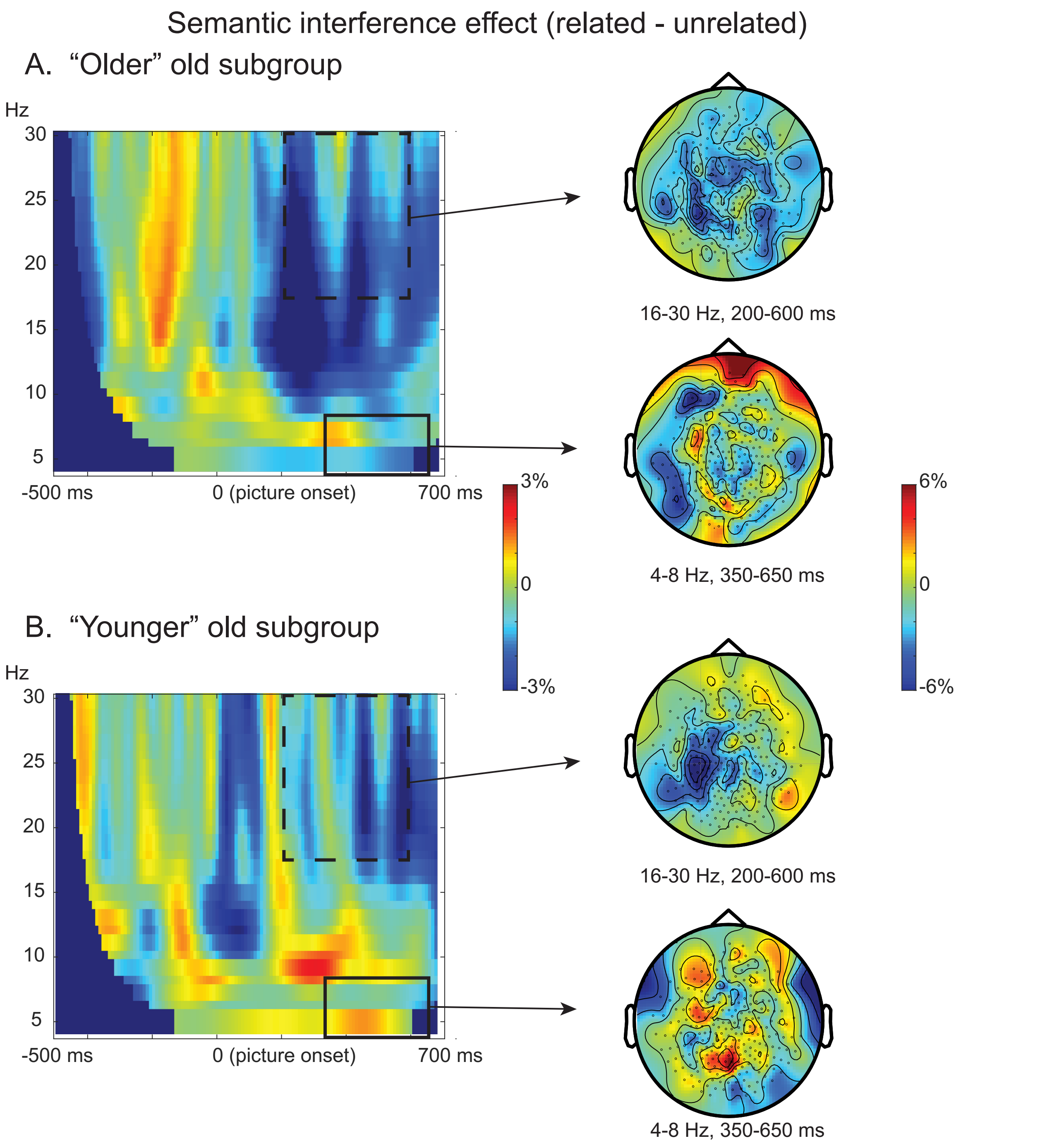

Figure S3. Semantic interference effects for the “older old” adults (top panel, N = 8, $mean_{Age}$ = 67.0, range = 61-72) and the “younger old adults” (bottom panel, N = 8, $mean_{Age}$ = 51.4, range = 46-59). Left: Stimulus-locked time-resolved spectrum of the contrast between related vs. unrelated conditions, averaged over all sensors. Right: Topography of the semantic contrast (i.e.,

related vs. unrelated) in the beta band (16-30 Hz) between 200 to 600 ms post picture onset, and in the theta band (4-8 Hz) between 350 to 650 ms post picture onset. Color bars indicate the relative power change between conditions.

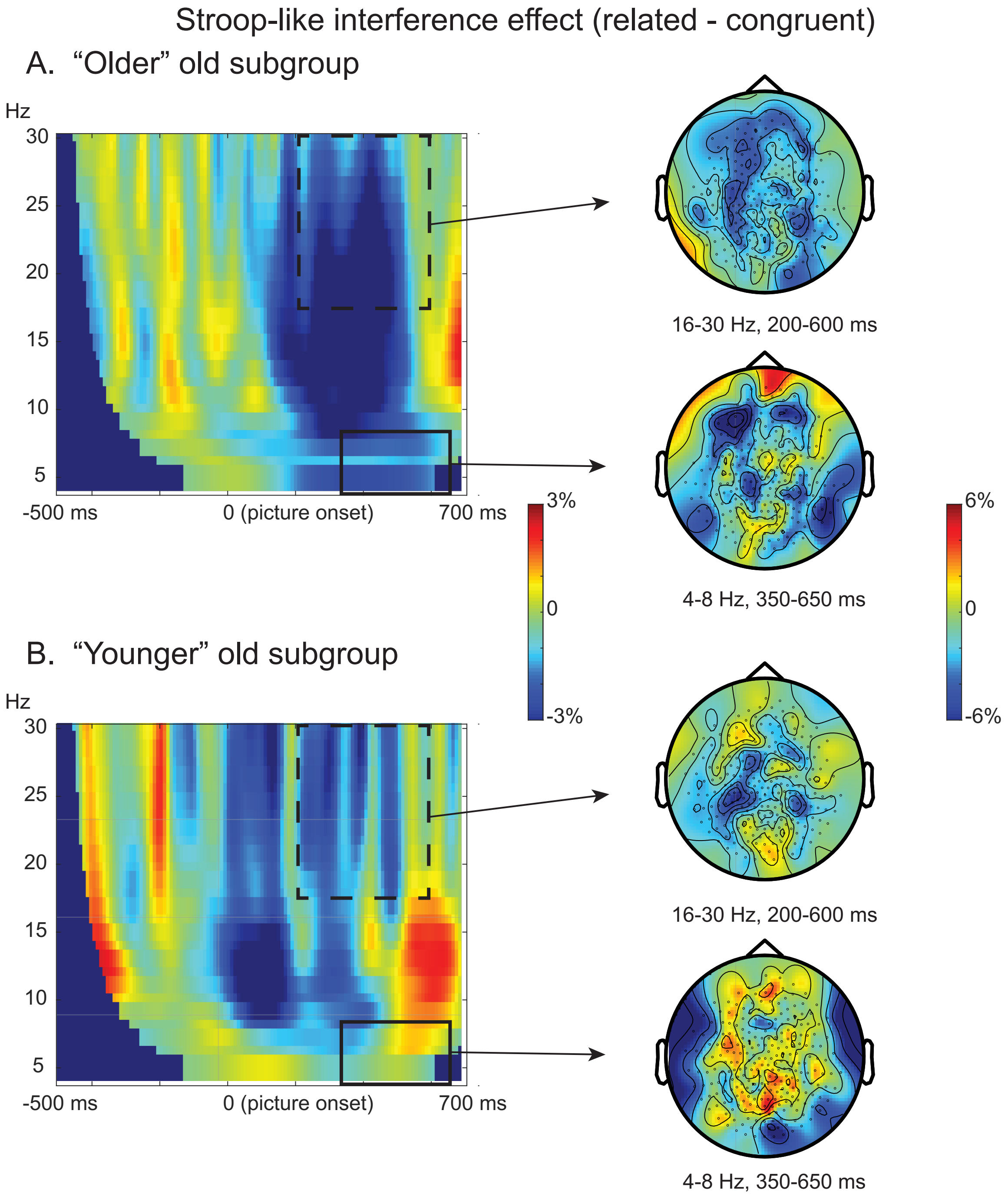

Figure S4. Stroop-like effects for the “older old” adults (top panel, N = 8, $mean_{Age}$ = 67.0, range = 61-72) and the “younger old” adults (bottom panel, N = 8, $mean_{Age}$ = 51.4, range = 46-59). Left: Stimulus-locked time-resolved spectrum of the contrast between related vs. congruent conditions, averaged over all sensors. Right: Topography of the stroop-like contrast (i.e., related

vs. congruent) in the beta band (16-30 Hz) between 200 to 600 ms post picture onset, and in the theta band (4-8 Hz) between 350 to 650 ms post picture onset. Color bars indicate the relative power change between conditions.
